# Ovulation Timing After Recombinant Human Chorionic Gonadotropin, Gonadorelin, or Buserelin Under Estrus-Triggered and Fixed-Time Treatment Protocols for Artificial Insemination in Sows

**DOI:** 10.3390/ani16203245

**Published:** 2026-10-15

**Authors:** Zihan Fang, Kexiong Liu, Xiaoling Xu, Yusheng Qin, Linli Xiao, Yuqing Song, Feng Ji, Heliang Zhang, Yan Liu, Jiahua Bai

**Affiliations:** 1 Institute of Animal Husbandry and Veterinary Medicine, Beijing Academy of Agriculture and Forestry Sciences, Beijing 100097, China; 2 College of Animal Science and Technology, Hebei Northern University, Zhangjiakou 075031, China

**Keywords:** sow, artificial insemination, fixed-time artificial insemination, ovulation induction, ovulation timing, recombinant human chorionic gonadotropin, gonadorelin, buserelin

## Abstract

Precise coordination between ovulation and insemination is essential for optimizing fixed-time artificial insemination (FTAI) in sows. This study compared ovulation timing and reproductive outcomes after treatment with recombinant human chorionic gonadotropin (rhCG), gonadorelin, or buserelin under estrus-triggered and fixed-time treatment protocols. A total of 285 sows received equine chorionic gonadotropin 24 h after weaning. In Trial 1, the ovulation-inducing treatments were administered at estrus onset, whereas in Trial 2 they were administered 72 h after equine chorionic gonadotropin. Ovulation was monitored by transabdominal ultrasonography. In Trial 1, no significant differences in ovulation timing or reproductive performance were detected among treatments. In Trial 2, no significant differences were detected among treatments in the proportion of sows with ovulation detected within 72 h (*p* = 0.847), the overall time-to-event distributions (log-rank *p* = 0.790), or the variability in detected ovulation timing (Brown–Forsythe *p* = 0.884). Among rhCG-treated sows in which ovulation was detected, all observed ovulations occurred between 37 and 54 h after treatment. No statistically significant differences in reproductive outcomes were detected among treatments. These findings provide new information on the temporal ovulatory response to rhCG under fixed-time treatment and may inform further refinement of insemination timing in sow FTAI protocols.

## Simple Summary

Fixed-time artificial insemination is most effective when insemination is closely aligned with ovulation. We compared gonadorelin, buserelin, and recombinant human chorionic gonadotropin (rhCG) in sows treated either at estrus onset or at a fixed time after equine chorionic gonadotropin (eCG). When treatment was given at estrus onset, no significant treatment differences in ovulation timing were detected. Under the fixed-time schedule, among rhCG-treated sows in which ovulation was detected, all observed ovulations occurred between 37 and 54 h after treatment. No statistically significant differences in reproductive outcomes were detected among treatments. This observed timing pattern warrants further evaluation for its potential relevance to fixed-time insemination.

## 1. Introduction

Fixed-time artificial insemination (FTAI) is increasingly used to simplify breeding management in pigs by reducing dependence on precise estrus detection and repeated inseminations. Under field conditions, FTAI protocols generally combine post-weaning hormonal stimulation or synchronization with an ovulation-inducing treatment, followed by one or more inseminations at predetermined times [1,2,3,4]. However, the success of FTAI depends strongly on the temporal relationship between insemination and ovulation [5,6,7]. Considerable variation in ovulation timing among sows can prolong the herd-level ovulation window and make it more difficult to align insemination with the optimal fertilization period [8,9]. Therefore, obtaining a more concentrated and clearly defined ovulation window is an important prerequisite for improving the precision and efficiency of FTAI programs.

Ovulation is commonly induced in synchronized gilts and sows using gonadotropin-releasing hormone (GnRH) analogues [10]. Gonadorelin is widely used for this purpose, whereas buserelin has greater stability and biological activity [10,11,12,13]. Studies using buserelin have demonstrated effective ovulation induction and its application in fixed-time insemination [14,15,16,17,18], with ovulation occurring predominantly within approximately 32–44 h after treatment [15]. Gonadorelin-induced ovulation has generally been reported at approximately 48 h after treatment [18]. Recombinant human chorionic gonadotropin (rhCG) provides an alternative approach by directly activating ovarian LH/CG receptors and bypassing the pituitary LH-release step required for GnRH analogues, which may result in a different temporal ovulatory response [19,20]. Conventional hCG has been used to synchronize ovulation in pigs, with ovulation commonly occurring approximately 38–48 h after treatment [21,22,23]. Recent studies have evaluated recombinant gonadotropin preparations in porcine reproductive management [24,25,26], but detailed information on the injection-to-ovulation interval after rhCG remains limited.

Our previous work showed that an FTAI protocol incorporating rhCG could achieve satisfactory reproductive performance compared with conventional equine chorionic gonadotropin (eCG)/GnRH-based protocols [26]. However, reproductive outcomes alone do not reveal how different ovulation-inducing preparations influence the temporal pattern of ovulation. To our knowledge, no previous study has systematically compared rhCG with commonly used GnRH analogues under both estrus-triggered and fixed-time treatment schedules in sows using serial ultrasonographic monitoring of ovulation. Such information is important for determining whether a given ovulation-induction strategy can provide a sufficiently defined ovulation window for more precise scheduling of insemination.

We therefore compared gonadorelin, buserelin, and rhCG under two treatment schedules: administration at estrus onset and administration at a fixed interval after eCG. Serial transabdominal ultrasonography was used to characterize ovulation timing, and reproductive outcomes were recorded in parallel. This design allowed us to determine whether the three preparations differed in their temporal patterns of ovulation and whether any such differences were accompanied by changes in fertility. We hypothesized that, owing to its direct action on ovarian LH/CG receptors, rhCG would produce a more temporally concentrated ovulatory response than gonadorelin or buserelin under both estrus-triggered and fixed-time treatment protocols.

## 2. Materials and Methods

### 2.1. Hormone Preparations

Altrenogest oral solution (Yunlibao, 4 mg/mL), eCG (Ailisheng, 1000 IU/vial), gonadorelin for injection (Shengyuan, 100, 100 μg/vial), and buserelin injection (Annuoqi, 4 μg/mL) were obtained from Ningbo Sansheng Biotechnology Co., Ltd. (Ningbo, China). Altrenogest was administered orally, whereas eCG, gonadorelin, and buserelin were administered intramuscularly. rhCG (Yiqipai, 2000 IU/vial) was supplied by Beijing Weijiexin Biotechnology Co., Ltd. (Beijing, China) and administered intramuscularly. 

### 2.2. Animals and Animal Management

The study was conducted at a commercial sow farm in Beijing, China, from May to July 2025. A total of 285 clinically healthy Landrace × Large White crossbred sows (parities 2–3; approximately 250 kg body weight) were enrolled. The farm operated a batch-production system with predetermined farrowing and weaning dates, and only sows with a 28-day lactation period were included to eliminate variation in lactation length. 

According to routine farm management, sows were transferred to the farrowing house on day 113 of gestation and received 4.2 kg feed/day until farrowing. Feed allowance was then gradually increased from 2.5 to 5.0 kg/day during the first 14 days of lactation. After weaning, sows were transferred to individual gestation crates and fed 3.6 kg/day from insemination to day 30 of gestation, 2.2 kg/day from days 31–90, and 4.2 kg/day thereafter. Water was available ad libitum, and all sows received the same diet and feed allowance within each reproductive stage, regardless of parity (Table 1).

### 2.3. Experimental Design 

**Figure 1:**
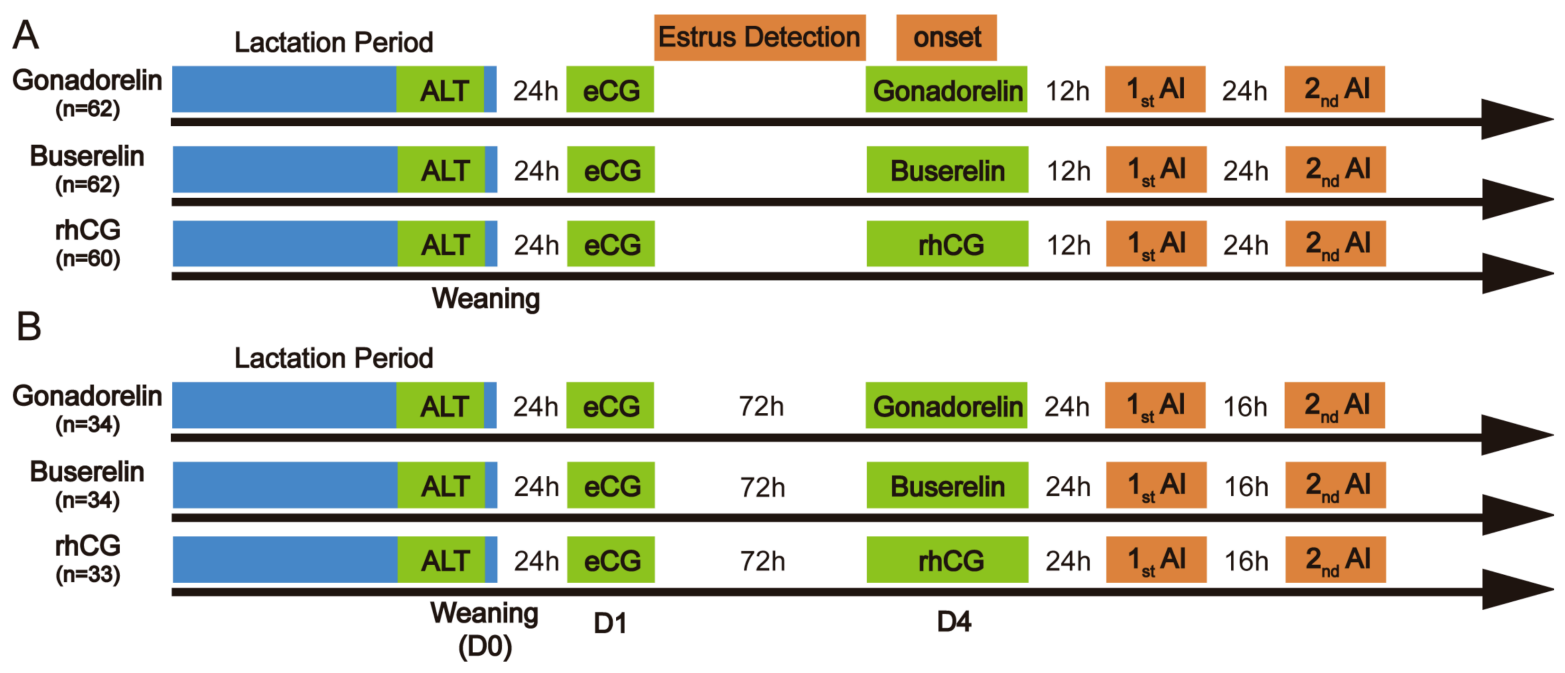
Schematic of experimental design with weaning sows. (**A**) Schematic of experimental design in trial 1. (**B**) Schematic of experimental design in trial 2.

All sows received ALT orally at 20 mg per sow per day from day 17 of lactation until the day before weaning (11 consecutive days) to improve post-weaning estrus synchronization [27]. Two trials were then conducted according to the timing of the ovulation-inducing injection (Figure 1). Within each trial, sows were ranked according to parity and sequentially allocated to the three treatment groups to achieve a similar parity distribution among groups. Group allocation was completed before administration of the ovulation-inducing treatments and before collection of treatment-related outcome data. All enrolled sows were retained in the reproductive and time-to-event analyses; descriptive ovulation-time distributions were calculated only among sows in which ovulation was detected.

#### 2.3.1. Trial 1

In Trial 1 (Figure 1A), all sows received 1000 IU eCG 24 h after weaning. At the onset of estrus, they received a single injection of either 100 μg gonadorelin (*n* = 62), 10 μg buserelin (*n* = 62), or 200 IU rhCG (*n* = 60). Insemination was performed 12 h after estrus onset and treatment administration and was repeated 24 h later. Notably, all sows in this trial were confirmed to be in estrus prior to treatment with ovulation-inducing preparations.

#### 2.3.2. Trial 2

In Trial 2 (Figure 1B), all sows received 1000 IU eCG 24 h after weaning, and the same three preparations (100 μg gonadorelin, *n* = 34; 10 μg buserelin, *n* = 34; 200 IU rhCG, *n* = 33) were injected 72 h later. Insemination was performed 24 h after administration of the ovulation-inducing treatment and was repeated 16 h later (40 h after treatment). 

### 2.4. Ultrasound Analysis

Transabdominal ultrasonography was performed with a Honda HS-1600 V system (Honda Electronics, Tokyo, Japan) equipped with a 5.0 MHz sector probe to precisely determine ovulation timing in sows. All ultrasonographic examinations were performed by the same experienced operator, who was blinded to treatment allocation throughout the study. In Trial 1, all sows were examined three times daily at 07:00, 15:00, and 23:00, beginning at estrus onset and continuing until ovulation was detected. In Trial 2, ultrasonographic examinations began on day 2 after eCG administration and were performed at the same time points until ovulation was detected or until 72 h after treatment. Ovulation was determined from changes in the number of large follicles between consecutive examinations, consistent with previously described ultrasonographic criteria [28]. When numerous large follicles were present at one examination but were no longer visible at the subsequent examination, the ovulation time was assigned as the midpoint between the two examinations. When the number of large follicles was markedly reduced at the subsequent examination but a few follicles remained, ovulation was considered to be in progress, and the time of that examination was recorded as the ovulation time. When ovulation was not detected within 72 h after treatment, the ovulation time was considered undetermined within the monitoring period.

### 2.5. Estrous Detection and Artificial Insemination 

In Trial 1, estrus detection was conducted twice daily in the presence of a sexually mature boar, commencing on the day of weaning. A technician applied fence-line boar contact together with the back-pressure test and recorded the standing reflex. The onset of estrus was recorded for each sow. In Trial 2, estrus detection was not performed because both the ovulation-inducing treatment and inseminations were scheduled at predetermined times irrespective of estrus expression. Each post-cervical artificial insemination (PCAI) dose contained 1.5 × 10^9^ spermatozoa in a total volume of 80 mL and was stored at 17 °C before use. Only semen with sperm motility >70% and abnormal spermatozoa <20% was used. Semen from 10 boars was used in Trial 1 and semen from 5 boars in Trial 2; within each trial, semen was pooled before preparation of the insemination doses and used across all three treatment groups. Pregnancy was diagnosed by transabdominal ultrasonography on day 30 after insemination.

### 2.6. Statistical Analysis

Data from Trials 1 and 2 were analyzed separately using SPSS version 24.0 (IBM Corp., Armonk, NY, USA), with the individual sow as the experimental unit. Normality was assessed using the Shapiro–Wilk test. Parity, total pigs born, and pigs born alive were analyzed by one-way ANOVA, whereas pregnancy rate, gestation farrowing rate, service farrowing rate, and the proportion of sows with detected ovulation were analyzed using Pearson’s chi-square test. Time to detected ovulation was analyzed using the Kaplan–Meier method and log-rank test; in Trial 2, sows without detected ovulation were right-censored at 72 h. Among sows with detected ovulation, dispersion of ovulation times was compared using the Brown–Forsythe test, and descriptive interval distributions used these sows as the denominator. Statistical significance was set at *p* < 0.05. 

## 3. Results

### 3.1. Ovulation Dynamics Following Treatment at Estrous Onset (Trial 1)

**Figure 2:**
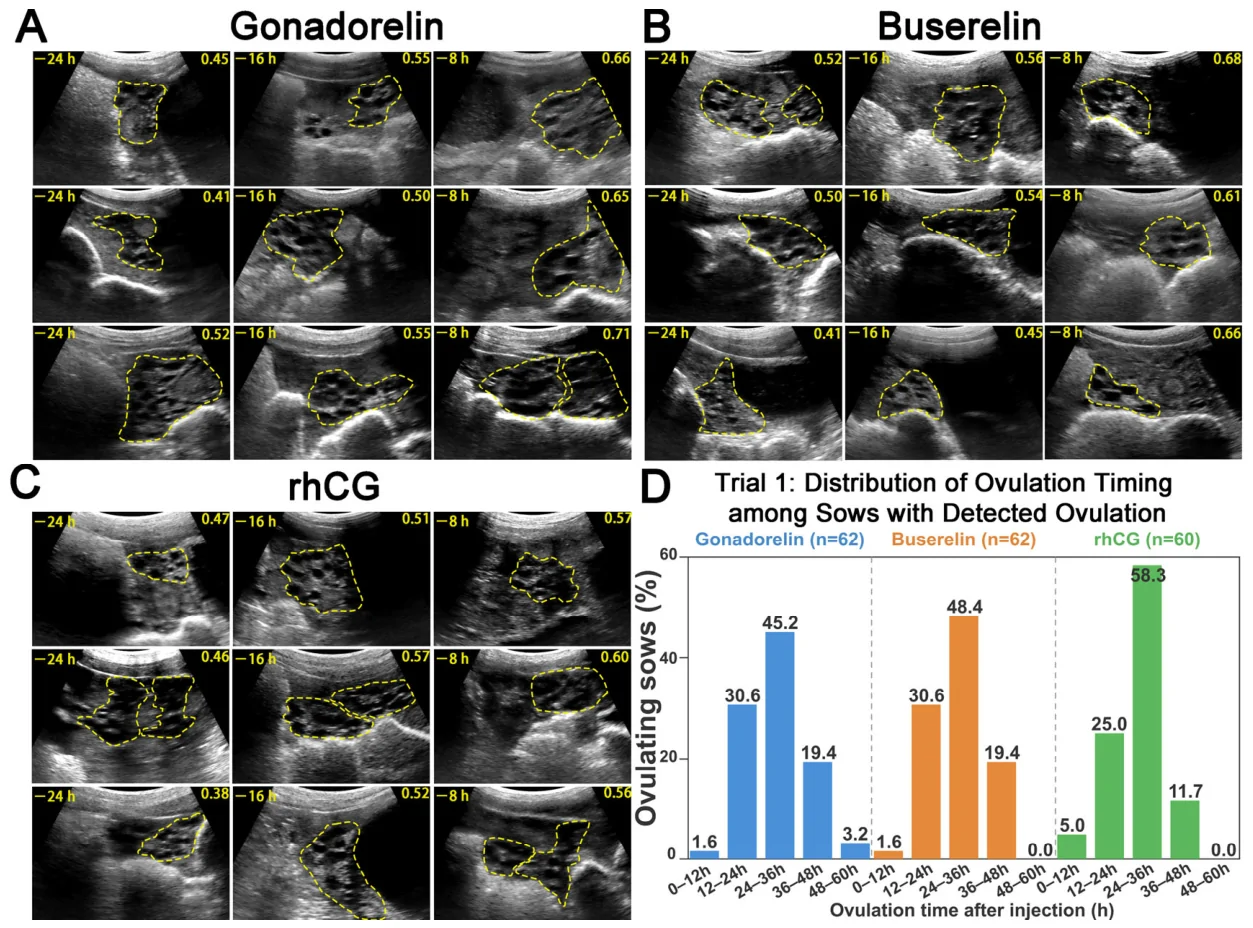
Follicular development before ovulation in Trial 1. Representative ultrasonographic images of pre-ovulatory follicles (8–24 h before ovulation, (**A**–**C**)) and the distribution of ovulation timing among sows (**D**). The dotted line delineates the ovary. The upper left corner of each image represents the time and the upper right corner represents the average follicle diameter (cm) at that time point.

Figure 2A–C show representative ultrasonographic images of follicles before ovulation. When the ovulation-inducing treatments were administered at estrus onset, the highest proportion of sows in each group ovulated during the 24–36 h interval after treatment (Figure 2D). The proportions were 58.3% for rhCG, 48.4% for buserelin, and 45.2% for gonadorelin. A small proportion of gonadorelin-treated sows (3.2%) ovulated after 48 h, whereas no ovulations occurred after 48 h in the buserelin or rhCG groups. Time-to-event analysis showed no significant difference in time to detected ovulation among treatments (log-rank χ^2^ = 1.850, *p* = 0.397). 

### 3.2. Reproductive Performance Following Treatment at Estrous Onset (Trial 1)

No statistically significant differences were detected among treatments in pregnancy rate, gestation farrowing rate, service farrowing rate, total pigs born, or pigs born alive (Table 2; *p* = 0.23, 0.20, 0.60, 0.30, and 0.63, respectively).

### 3.3. Ovulation Dynamics Following Fixed-Time Treatment (Trial 2)

**Figure 3:**
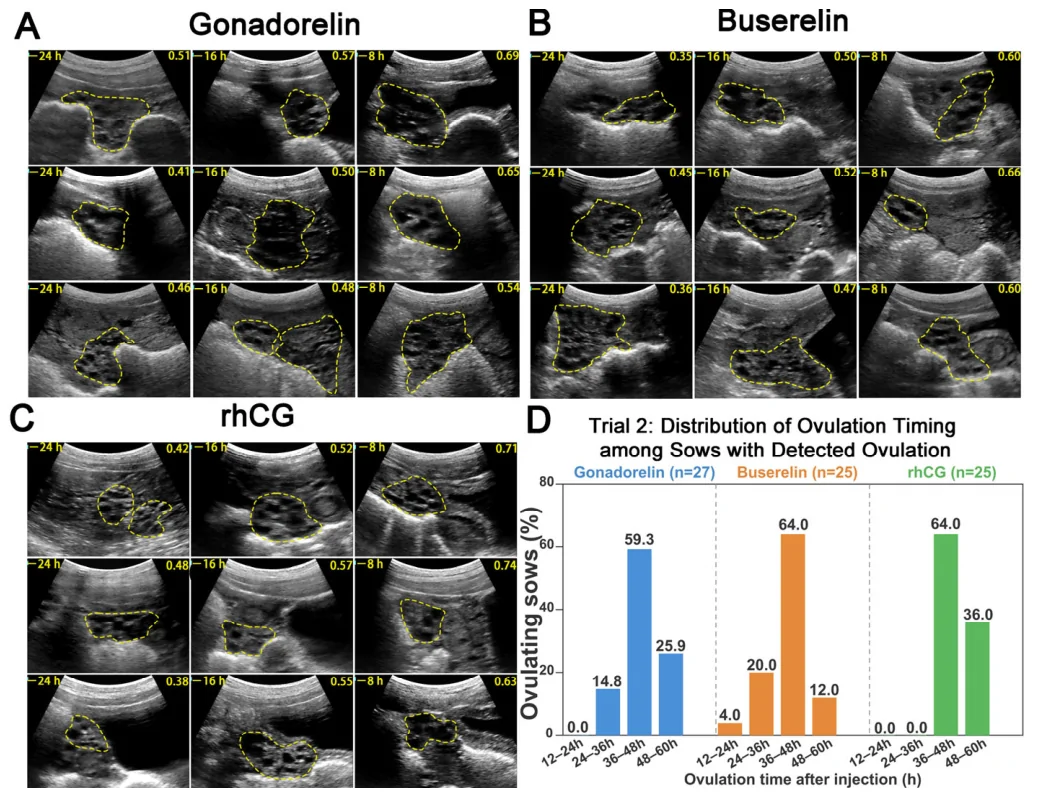
Follicular development before ovulation in Trial 2. Representative ultrasonographic images of pre-ovulatory follicles (8–24 h before ovulation, (**A**–**C**)) and the distribution of ovulation timing among sows in which ovulation was detected during the monitoring period (**D**). The dotted line delineates the ovary. The upper left corner of each image represents the time and the upper right corner represents the average follicle diameter (cm) at that time point.

Figure 3A–C show representative ultrasonographic images of follicles before ovulation. Within 72 h after treatment, ovulation was detected in 79.4% (27/34), 73.5% (25/34), and 75.8% (25/33) of sows treated with gonadorelin, buserelin, and rhCG, respectively, with no significant difference among treatments (*p* = 0.847). The time-to-event analysis showed no significant difference in the time to detected ovulation among treatments (log-rank *p* = 0.790). Among sows with detected ovulation, the variability in ovulation timing did not differ significantly among treatments (Brown–Forsythe *p* = 0.884). The highest proportion of detected ovulations in all three groups occurred during the 36–48 h interval after treatment (Figure 3D). In the rhCG group, all detected ovulations occurred between 37 and 54 h after treatment.

### 3.4. Reproductive Performance Following Fixed-Time Treatment (Trial 2)

No statistically significant differences were detected among treatments in pregnancy rate, gestation farrowing rate, service farrowing rate, total pigs born, or pigs born alive (Table 3; *p* = 0.83, 0.17, 0.53, 0.31, and 0.46, respectively).

## 4. Discussion

No significant differences in time to detected ovulation were observed among treatments under either protocol. Under the fixed-time protocol, neither the proportion of sows with ovulation detected within 72 h nor the overall time-to-event distributions differed among treatments. Among rhCG-treated sows in which ovulation was detected, all observed ovulations occurred between 37 and 54 h; however, ovulation-time variability did not differ significantly among treatments.

The contrast between the two trials may reflect where the treatments act within the ovulatory axis. rhCG directly activates ovarian LH/CG receptors [19,20], whereas gonadorelin and buserelin depend on pituitary LH release [10,11]. The response to GnRH stimulation is influenced by the endocrine and follicular status of the sow [29,30,31], while the greater stability and biological activity of buserelin may provide a more sustained GnRH stimulus [11,12,13,14,15]. Previous studies have likewise shown that the timing of ovulation induction relative to reproductive status is important for the subsequent ovulatory response and fixed-time insemination [31,32,33]. However, circulating LH was not measured; therefore, these mechanisms remain physiological interpretations rather than findings directly demonstrated in this study. 

The timing observed in Trial 2 is also relevant to the insemination schedule. The same insemination schedule, at 24 and 40 h after treatment, was applied to all three treatment groups. Sperm deposited before ovulation can remain capable of fertilization for several hours in the female reproductive tract [5,6,7,8,9], providing temporal coverage of the observed ovulation period. Previous studies have shown that fertility is strongly influenced by the interval between insemination and ovulation [7,8,9]. However, because alternative insemination schedules were not directly compared, the present study cannot determine whether a different schedule, including a single fixed-time insemination, would maintain or improve reproductive performance, and this possibility requires direct testing [34].

No statistically significant differences in reproductive outcomes were detected among treatment groups in either trial. This is consistent with earlier work in pigs. De Jong et al. [35] found that the GnRH analogue Maprelin improved estrus expression and synchronization in gilts and sows, but farrowing rate and litter size were not uniformly better across parity groups. Another GnRH analogue, Peforelin, gave only modest gains that were mostly not significant [36]. Degenstein et al. [37] demonstrated that pLH synchronized ovulation in cyclic gilts without significantly affecting embryo quality. Taken together, these findings indicate that a more clearly defined ovulatory response does not necessarily translate into improved reproductive performance, as fertility outcomes are also influenced by other biological and management factors.

Several limitations should be considered. Trial 2 included only 33–34 sows per treatment and no formal a priori sample-size calculation was performed, which may have limited the power to detect modest effects. Ovulation was not detected within 72 h in 7/34, 9/34, and 8/33 sows in the gonadorelin, buserelin, and rhCG groups, respectively; the subsequent pregnancy data indicate that at least some of these sows ovulated after the monitoring window, suggesting that the observed ranges may underestimate the full distribution of ovulation timing. Circulating LH was not measured, and the use of an ALT-based synchronization protocol and sows only may limit extrapolation to other management systems or gilts.

## 5. Conclusions

No significant treatment differences in time to detected ovulation were observed under either protocol. Under the fixed-time protocol, all detected ovulations in the rhCG group occurred between 37 and 54 h, although ovulation-time variability did not differ significantly among treatments. No statistically significant differences in reproductive outcomes were detected, and the practical relevance of the observed rhCG timing pattern for insemination scheduling requires further investigation.

## Figures and Tables

**Table 1:** Ingredients and nutrient composition of the experimental diets.

Item	Lactating Sows	Gestating Sows
Ingredient	Ingredient proportion (%)	
fish meal	3	-
Soybean hulls	-	6.96
2% Premixed feed	2	2
Wheat bran	3	11.6
Corn grain	56.01	47.11
Soybean oil	2	0.5
Soybean meal	18.83	11.8
Stone powder	1.32	1.21
CaHPO_4_	0.84	1.32
Wheat middling	5	12.5
Beet meal granules	-	5
Flour	8	-
Total	100	100
Nutritive index		
Crude protein (%)	17.64	13.97
Digestible energy (Kcal/kg)	3452	3135
Crude fat (%)	4.98	3.23
Crude ash (%)	6.1	6.82
Crude fibers (%)	2.98	6.31
Ca (%)	0.85	0.85
Lysine (%)	1.13	0.66
Vitamin A (IU/kg)	6996	11872
Vitamin D3 (IU/kg)	2400	3200
Vitamin E (mg/kg)	84	96.49
Vitamin K3 (mg/kg)	7.8	7.8
Vitamin B12 (mg/kg)	60	40
Vitamin B1 (mg/kg)	1.8	1.8
Vitamin B2 (mg/kg)	6.4	6.4
Vitamin B6 (mg/kg)	3.33	3.33
Pantothenic acid (mg/kg)	21.78	21.78
Niacin (mg/kg)	35.64	35.64
Choline chloride (mg/kg)	249.6	585
Folacin (mg/kg)	4.36	4.36
Biotin (mg/kg)	0.44	0.44
Mn (mg/kg)	29.58	36.7
Fe (mg/kg)	265	288
Zn (mg/kg)	79.75	92.5
Cu (mg/kg)	17.8	22.24
I (mg/kg)	0.1	0.11
Se (mg/kg)	0.44	0.46

**Table 2:** The reproductive performance of sows in Trial 1.

Terms	Gonadorelin	Buserelin	rhCG	SEM	*p*-Value
*n*	62	62	60	-	-
Parity	2.26	2.25	2.16	0.06	0.21
Weaning-to-estrus interval (h)	117.8	119.0	118.8	1.28	0.92
Pregnant rate ^1^ (%)	88.7 (55/62)	96.8 (60/62)	91.7 (55/60)	-	0.23
Gestation farrowing rate ^2^ (%)	98.2 (54/55)	95.0 (57/60)	100.0 (55/55)	-	0.20
Service farrowing rate ^3^ (%)	87.1 (54/62)	91.9 (57/62)	91.7 (55/60)	-	0.60
Total pigs born/litter (mean)	14.35	14.02	14.87	0.23	0.30
Pigs born alive/litter (mean)	11.46	11.70	12.04	0.24	0.63

^1^ Pregnant rate = no. of pregnant sows/no. of all treated sows. ^2^ Gestation farrowing rate = no. of farrowing sows/no. of pregnant sows. ^3^ Service farrowing rate = no. of farrowing sows/no. of all treated sows.

**Table 3:** The reproductive performance of sows in Trial 2.

Terms	Gonadorelin	Buserelin	rhCG	SEM	*p*-Value
*n*	34	34	33	-	-
Parity	2.50	2.33	2.67	0.08	0.51
Pregnant rate ^1^ (%)	82.4 (28/34)	76.5 (26/34)	78.8 (26/33)	-	0.83
Gestation farrowing rate ^2^ (%)	78.6 (22/28)	84.6 (22/26)	96.2 (25/26)	-	0.17
Service farrowing rate ^3^ (%)	64.7 (22/34)	64.7 (22/34)	75.8 (25/33)	-	0.53
Total pigs born/litter (mean)	13.71	12.63	14.00	0.40	0.31
Pigs born alive/litter (mean)	11.86	11.00	12.17	0.39	0.46

^1^ Pregnant rate = no. of pregnant sows/no. of all treated sows. ^2^ Gestation farrowing rate = no. of farrowing sows/no. of pregnant sows. ^3^ Service farrowing rate = no. of farrowing sows/no. of all treated sows.

## Data Availability

None of the data were deposited in an official repository. All data generated during the study are available from the corresponding author by request.

## Abbreviations

The following abbreviations are used in this manuscript: AI: artificial insemination, ALT: altrenogest, eCG: equine chorionic gonadotropin, FTAI: fixed-time artificial insemination, GnRH: gonadotropin-releasing hormone, hCG: human chorionic gonadotropin, IU: international unit, PCAI: post-cervical artificial insemination, LH: luteinizing hormone, rhCG: recombinant human chorionic gonadotropin.
